# Johnson’s Cyclopedia

**Published:** 1889-07

**Authors:** 


Read the advertisement of JOHNSON'S UNIVERSAL CYCLOPEDIA. This is truly a great work. Epecially adapted as a work of reference for medical men and progressive men of every class, who may desire information upon any subject.

